# Effects of dapagliflozin on serum and urinary uric acid levels in patients with type 2 diabetes: a prospective pilot trial

**DOI:** 10.1186/s13098-020-00600-9

**Published:** 2020-10-27

**Authors:** Tao Yuan, Shixuan Liu, Yingyue Dong, Yong Fu, Yan Tang, Weigang Zhao

**Affiliations:** 1 Department of Endocrinology, Key Laboratory of Endocrinology of National Health Commission, Peking Union Medical College Hospital, Peking Union Medical College and Chinese Academy of Medical Sciences, Shuaifuyuan Street No.1, Dongcheng District, Beijing, 100730 China; 2 Department of Pharmacology, Peking Union Medical College Hospital, Peking Union Medical College and Chinese Academy of Medical Sciences, Beijing, China

**Keywords:** Serum uric acid, Fractional excretion of uric acid, Dapagliflozin, Type 2 diabetes mellitus, Islet β-cell function, SGLT2 inhibitors

## Abstract

**Background:**

We aimed to evaluate the effects of short-term therapy with dapagliflozin on serum uric acid (SUA) and urinary uric acid (UUA) levels in patients with type 2 diabetes.

**Methods:**

In this prospective pilot trial, 8 patients with type 2 diabetes mellitus were assigned to the treatment group with dapagliflozin 10 mg once daily for one week, and 7 subjects with normal glucose tolerance were recruited into the control group. Data of anthropometric measurements, SUA, 24-h UUA, fractional excretion of UA (FEUA), serum lipid parameters and 3-h oral glucose tolerance test (OGTT) were collected in both treatment and control groups; all examinations were repeated after treatment. The area under the curve of glucose (AUC_Glu_) was calculated to reflect the general glucose levels, while insulin resistance and islet β-cell function were reflected by indexes calculated according to the data obtained from the OGTT.

**Results:**

The weight and serum lipid parameters showed no differences before and after treatment with dapagliflozin for one week. We found SUA levels decreased from 347.75 ± 7.75 μmol/L before treatment to 273.25 ± 43.18 μmol/L after treatment, with a statistically significant difference (P = 0.001) and was accompanied by a significant increase in FEUA from 0.009 to 0.029 (P = 0.035); there was a linear correlation between SUA and FEUA levels. Glucose control, insulin sensitivity and islet β-cell function were improved to a certain extent. We also found a positive correlation between the decrease in glucose levels and the improvement in islet β-cell function.

**Conclusions:**

The SUA-lowering effect of dapagliflozin could be driven by increasing UA excretion within one week of treatment, and a certain degree of improvement in glucose levels and islet β-cell function were observed.

*Trial registration* ClinicalTrials.gov identifier, NCT04014192. Registered 12 July 2019, https://www.clinicaltrials.gov/ct2/show/NCT04014192:term=NCT04014192&draw=2&rank=1. Yes.

## Background

Type 2 diabetes mellitus (T2DM) is a complex metabolic disease, which is characterized by insulin resistance of different insulin target tissues (including liver, adipose tissue, and skeletal muscle) and insufficient insulin secretion by pancreatic β-cells, as well as associated with other metabolic diseases, including obesity, dyslipidemia, hyperuricemia and non-alcoholic fatty liver disease [1]. Therefore, ideal hypoglycemic drugs were considered to not only improve glycemic control but also to benefit combined metabolic disorders. Under these circumstances, there are many new hypoglycemic drugs that have emerged in recent years, including sodium-glucose cotransporter-2 (SGLT2) inhibitors. SGLT2 is an active glucose transporter located in the early proximal renal tubule and accounts for 90% of glucose reabsorption by the kidneys, the expression of which is increased in both animal models of diabetes and patients with diabetes. SGLT2 inhibitors exert hypoglycemic effects by increasing urine glucose excretion in an insulin-independent way [2]. Currently, a variety of SGLT2 inhibitors have been marketed around the world with gradually increasing clinical application.

Studies on beneficial effects other than the hypoglycemic effects of SGLT2 inhibitors have attracted increasing attention, including decreasing risk of cardiovascular diseases, heart failure, and kidney diseases, as well as reducing serum uric acid (SUA) levels and events related to gout flares among patients with T2DM [3–5]. In addition, a few studies have found that SGLT2 inhibitors can play a beneficial role in improving insulin resistance and islet β-cell function [6–8], as well as help to reduce weight, increase lipolysis of adipose tissue and reduce fat production after long-term treatment. However, the long-term treatment period adopted by most previous studies makes it difficult to distinguish whether the beneficial effects on islet β-cell function resulted from the improvement in glucotoxicity, or changes in weight and lipid metabolism.

In this study, we aimed to perform a one week study to evaluate effects of dapagliflozin on levels of SUA and urinary uric acid (UUA) and explore whether the improvement in glucotoxicity alone can benefit insulin resistance and islet β-cell function.

## Methods

### Participants

Eight subjects were recruited from patients with T2DM (the treatment group) in the clinic of the Endocrinology Department at Peking Union Medical College Hospital (PUMCH) from January 2019 to December 2019. These subjects were selected from individuals aged 18 to 70 (including 18 and 70-year-old individuals), whose glycated hemoglobin (HbA1c) was ≥ 7% and estimated glomerular filtration rate (eGFR) was ≥ 60 mL/min/1.73 m^2^ without contraindications to SGLT2 inhibitors. There were no limitations with respect to the duration of T2DM, gender and basic antidiabetic therapy. The eGFR was calculated using the Modification of Diet in Renal Disease (MDRD) formula [9, 10].

Exclusion criteria included a diagnosis of other types of diabetes mellitus (DM); unstable control of blood glucose (fasting blood glucose (FBG) > 11.1 mmol/L); acute complications of T2DM within the last 6 months; history of myocardial infarction or stroke within the last 6 months or existing severe cardiovascular disease and risk; abnormal liver function (i.e., serum alanine aminotransferase or aspartate aminotransferase 1.5 times higher than the upper limit of normal); severe hypertension defined as a systolic blood pressure ≥ 160 mmHg or diastolic blood pressure ≥ 90 mmHg with drug therapy or hypotension (resting seated blood pressure < 90/50 mmHg); severe anaemia; psychosis, alcohol dependence or history of drug abuse; lactating women; participation in other studies three months before the present trial; allergic constitution or allergic to a variety of drugs; and a judgement of ineligibility to participate by researchers for any other reasons.

Seven healthy volunteers with normal glucose tolerance (NGT) (2-h plasma glucose level < 7.8 mmol/L) and normal fasting glucose level (FBG < 5.6 mmol/L) were recruited as the control group. The diagnosis of NGT and normal fasting glucose level were based on the diagnostic criteria of the American Diabetes Association (ADA) [11].

This study was approved by the PUMCH Ethics Committee and followed the ethical standards of the responsible committee on human experimentation (institution and national) and with the Declaration of Helsinki (1964), as revised in 2013. All participants signed written informed consent voluntarily. The clinical trial number for this study is NCT04014192.

### Study design

**Fig. 1:**
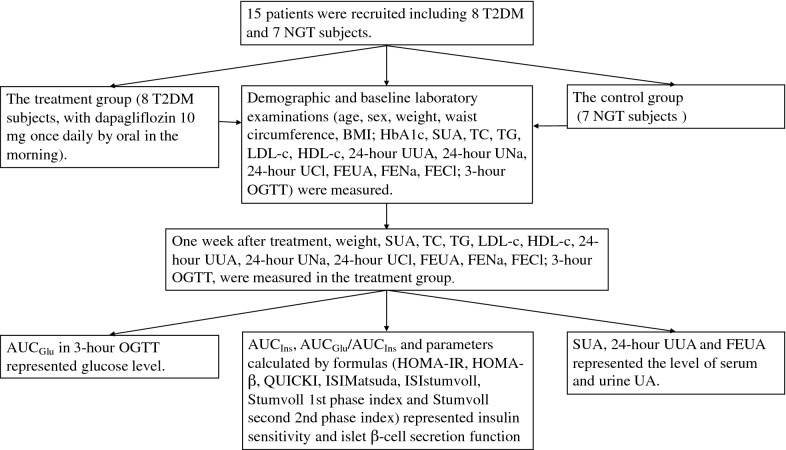
Clinical trial flow chart. T2DM, type 2 diabetes mellitus; NGT, normal glucose tolerance; BMI, body mass index; HbA1c, glycated hemoglobin; SUA, serum uric acid; TC, total cholesterol; TG, triglyceride; LDL-c, low-density lipoprotein cholesterol; HDL-c, high-density lipoprotein cholesterol; UUA, urinary uric acid; UNa, urinary sodium; UCl, urinary chloride; FEUA, fractional excretion of uric acid; FENa, fractional excretion of sodium; FECl, fractional excretion of chloride; OGTT, oral glucose tolerance test; AUC_Glu_, area under the curve of glucose; AUC_Ins_, area under the curve of insulin; HOMA-IR, homeostasis model assessment of insulin resistance; HOMA-β, homeostasis model assessment of β-cell function; QUICKI, quantitative insulin sensitivity check index; ISIstumvoll, insulin sensitivity index proposed by Stumvoll et al.; ISIMatsuda, insulin sensitivity index proposed by Matsuda et al.; 1st, first; 2nd, second

Subjects in the treatment group were treated with dapagliflozin 10 mg orally once daily in the morning for one week. All measurements of physical examinations and blood and urine samples were collected at baseline for all subjects in the two groups and repeated for subjects in the treatment group after one week of treatment. The clinical trial flow chart is shown in Fig. 1.

### Anthropometric measurements

Medical records were accessed for baseline information, including age, sex, weight, height, and waist circumference. Body mass index (BMI) was calculated as weight (kg) divided by the square of the height in metres (m^2^).

### Laboratory measurements

Laboratory parameters were collected after fasting for 8 to 12 h, including FBG, HbA1c, SUA, blood urea nitrogen, serum creatinine, serum total cholesterol (TC), high-density lipoprotein cholesterol (HDL-c), low-density lipoprotein cholesterol (LDL-c) and triglyceride (TG) levels.

The blood samples were collected from the forearm to assay the serum glucose, insulin and C-peptide levels at fasting time (0 min), and 30 min, 60 min, 120 min and 180 min after an oral 75 g anhydrous glucose load. The area under the curve of glucose (AUC_Glu_) was calculated to reflect the glucose levels. The Quantitative Insulin Sensitivity Check Index (QUICKI) [12], insulin sensitivity index proposed by Matsuda et al. (ISIMatsuda) [13], insulin sensitivity index proposed by Stumvoll et al. (ISIstumvoll) [14], the ratio of area under curve of glucose and insulin (AUC_Glu_/AUC_Ins_) [15] and homeostasis model assessment of insulin resistance (HOMA-IR) [16] were calculated to reflect insulin resistance. The homeostasis model assessment of β-cell function (HOMA-β) [16], area under the curve of insulin (AUC_Ins_), Stumvoll first (1st) phase index and Stumvoll second (2nd) phase index were calculated to reflect islet β-cell function [17].

The 24-h urinary samples were collected to assay the UUA, urinary sodium (UNa), urinary chloride (UCl), and urinary creatinine; the fractional excretions of UA (FEUA), sodium (FENa) and chloride (FECl) were calculated respectively.

Biochemical parameters were measured by a Beckman automatic biochemical analyser (AU5800, Beckman Coulter, CA, USA), and plasma insulin and C-peptide levels were measured by the ADVIA Centaur XP immunoassay system (Siemens, New York, USA).

### Outcome measures

The primary endpoint was changes in SUA levels from baseline to post-treatment. Additional outcomes included changes in 24-h UUA; FEUA; parameters of glucose, insulin resistance and islet β-cell function; weight; lipid parameters; and urinary parameters of sodium and chloride.

### Statistical analysis

Continuous variables were expressed as the mean ± standard deviation. Students' t test was used to compare differences between continuous variables in each group, and the continuous variables that failed the normality test were tested by the Wilcoxon rank sum test. A P-value less than 0.05 was considered statistically significant. Associations between variables were assessed using Pearson’s correlation coefficient. All statistical analyses were carried out using the statistical program SPSS (version 25, SPSS, Chicago, IL).

## Results

### Clinical characteristics of participants

Between the treatment group and control group, there were no statistically significant differences in genders, waist circumference, weight or BMI. FBG (P = 0.005), AUC_Glu_ (P < 0.001) in the 3-h oral glucose tolerance test (OGTT) and HbA1c (P = 0.002) were significantly higher in the treatment group compared with the control group, which was in accord with the characteristics of glucose metabolism in these two groups. SUA levels were higher in the treatment group compared with the control group.

In the treatment group, significantly lower SUA levels (P = 0.001) could be observed after taking dapagliflozin. The levels of AUC_Glu_ (P = 0.066) decreased after treatment, but without statistically significant difference, which might be due to the short period of treatment. Weight and lipid parameters also showed no statistically significant differences before and after treatment.

The baseline clinical characteristics between the two groups and before and after treatment with dapagliflozin are summarized in Table 1.

**Table 1:** Demographic and general laboratory blood parameters

Parameter	Control	Dapagliflozin 10 mg		P-value	
		Before	After	Con vs Bef	Bef vs Aft
Age (years)	41.71 ± 6.96	54.00 ± 9.61	–	0.011	–
Men, n (%)	4 (57.14)	5 (62.5)	–	0.833	–
Weight (kg)	71.57 ± 16.15	83 ± 9.46	66.76 ± 9.79	0.674	0.360
BMI (kg/m^2^)	25.16 ± 3.56	27.10 ± 2.49	–	0.549	–
Waist circumference (cm)	87.86 ± 12.14	100.00 ± 7.33	–	0.624	–
AUC_Glu_ (mmol/L·h)	20.02 ± 4.15	46.73 ± 7.49	42.66 ± 7.45	< 0.001	0.066
HbA1c (%)	5.43 ± 0.23	7.54 ± 1.42	–	0.002	–
FBG (mmol/L)	5.03 ± 0.55	7.43 ± 1.58	7.43 ± 0.97	0.005	1.000
FINS (μIU/mL)	9.75 ± 5.05	11.10 ± 4.26	11.49 ± 4.10	0.387	0.468
SUA (μmol/L)	283.57 ± 99.93	347.75 ± 32.53	273.25 ± 43.18	0.175	0.001
TC (mmol/L)	4.98 ± 0.57	5.35 ± 0.83	5.34 ± 0.90	0.376	0.947
TG (mmol/L)	1.69 ± 0.87	2.10 ± 0.98	1.71 ± 0.74	0.441	0.135
HDL-c (mmol/L)	1.36 ± 0.44	1.05 ± 0.13	1.09 ± 0.16	0.144	0.191
LDL-c (mmol/L)	3.04 ± 0.51	3.57 ± 0.79	3.72 ± 1.01	0.186	0.282

Con, control; Bef, before; Aft, after; BMI, body mass index; AUC, area under the curve; Glu, glucose; HbA1c, glycated hemoglobin; FBG, fasting blood glucose; FINS, fasting serum insulin; SUA, serum uric acid; TC, total cholesterol; TG, triglyceride; HDL-c, high-density lipoprotein cholesterol; LDL-c, low-density lipoprotein cholesterol

### Characteristics of urinary parameters

At baseline, the concentration of 24-h UUA and FEUA were both higher in the treatment group compared with the control group, which indicated a higher excretion of uric acid (UA) in subjects with T2DM.

**Fig. 2:**
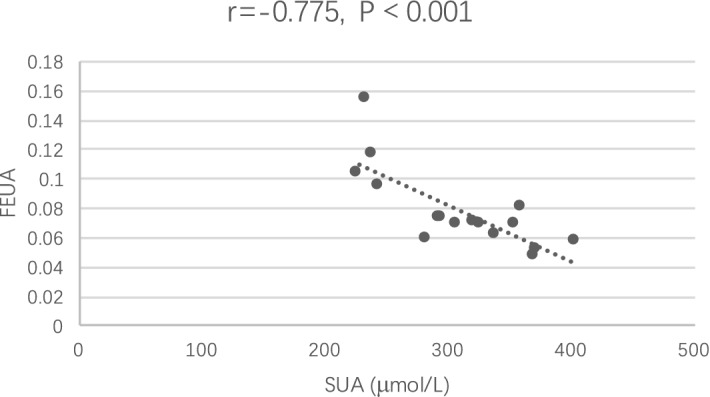
Correlation between SUA levels and FEUA levels before and after treatment. The relationship between the two variables was assessed using Pearson’s correlation coefficient. SUA, serum uric acid; FEUA, fractional excretion of uric acid

After treatment, both the FENa (P = 0.022) and FECl (P = 0.015) significantly increased, which is in line with the action mechanism of this class of drugs. Notably, the FEUA (P = 0.035) significantly increased after one week of treatment with dapagliflozin, and the SUA levels were inversely correlated with the FEUA levels (r = − 0.775, P < 0.001; Fig. 2). The concentrations of 24-h UNa, 24-h UCl and 24-h UUA increased, but showed no statistically significant differences.

**Fig. 3:**
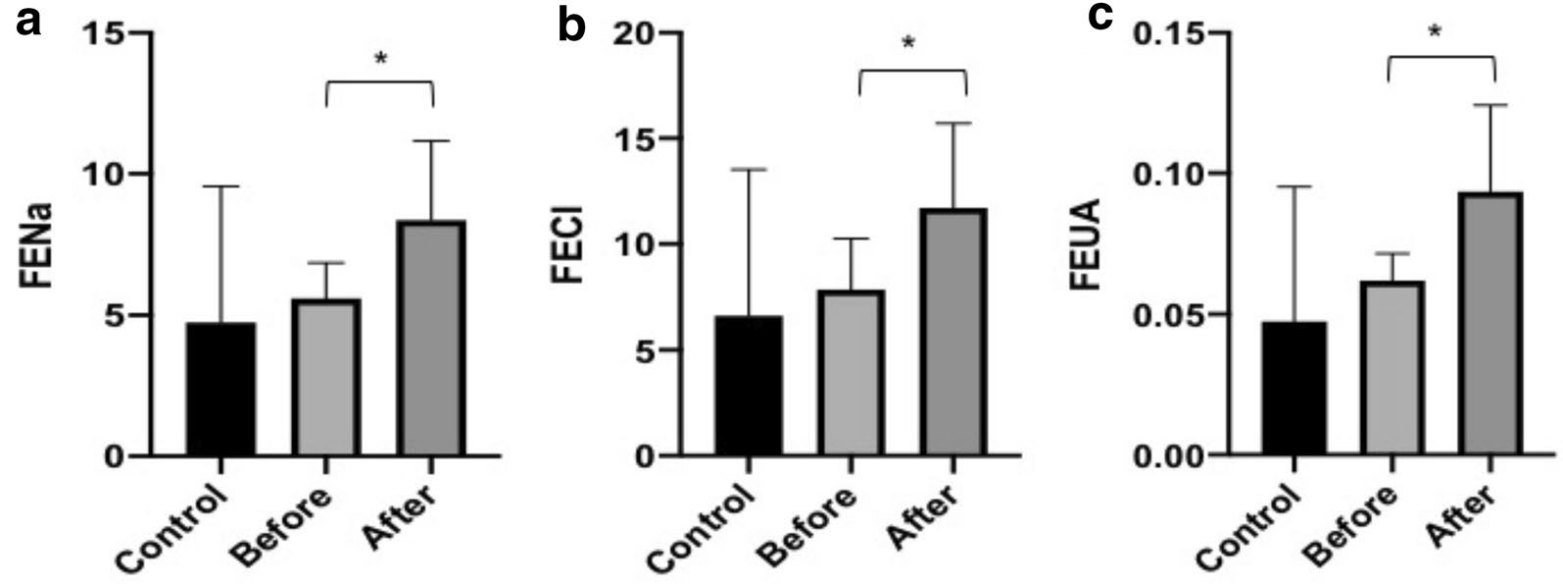
The comparison of fractional excretion of Na (**a**), Cl (**b**) and UA (**c**) in three groups. * represents statistical significance. FENa, fractional excretion of sodium; FECl, fractional excretion of chloride; FEUA, fractional excretion of uric acid

The changes of the urinary parameters in the control group and before and after taking dapagliflozin in the treatment group are presented in Table 2. It revealed changes in the levels of FENa, FECl and FEUA (Fig. 3a–c).

**Table 2:** Comparison of urinary parameters

Parameters	Control	Dapagliflozin 10 mg		P-value	
		Before	After	Con vs Bef	Bef vs Aft
24-h UUA (mmol)	3.26 ± 1.29	3.53 ± 0.61	3.79 ± 1.05	0.626	0.567
24-h UNa (mmol)	174.57 ± 46.13	126.18 ± 18.89	129.61 ± 27.34	0.025	0.057
24-h UCl (mmol)	167.71 ± 53.83	129.61 ± 27.34	177.00 ± 36.43	0.125	0.055
FEUA	0.047 ± 0.044	0.062 ± 0.009	0.093 ± 0.029	0.460	0.035
FENa	4.75 ± 4.46	5.58 ± 1.19	8.35 ± 2.63	0.672	0.022
FECl	6.63 ± 6.39	7.84 ± 2.27	11.71 ± 3.74	0.674	0.015

Con, control; Bef, before; Aft, after; UUA, urinary uric acid; UNa, urinary sodium; UCl, urinary chloride; FEUA, fractional excretion of uric acid; FENa, fractional excretion of sodium; FECl, fractional excretion of chloride

### Comparison of glycemic metabolism and islet β-cell function

The comparison of insulin resistance parameters, including HOMA-IR, QUICKI, ISIMatsuda, ISIstumvoll and AUC_Glu_/AUC_Ins_ revealed improvement in insulin sensitivity. Additionally, the islet β-cell function identified by HOMA-β, Stumvoll 1st phase index and Stumvoll 2nd phase index improved. The AUC_Ins_ decreased after taking dapagliflozin.

**Fig. 4:**
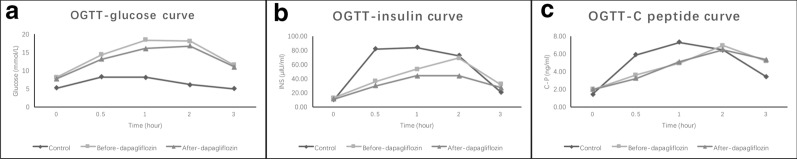
The curve of serum glucose (**a**), insulin (**b**) and C-peptide (**c**) levels in 3-h OGTT in three groups. OGTT, oral glucose tolerance test

The comparison of islet β-cell function between the control and treatment groups and changes before and after taking dapagliflozin are shown in Table 3. The curves of serum glucose (Fig. 4a), insulin (4b) and C-peptide (4c) levels in the 3-h OGTT are shown in Fig. 4.

**Table 3:** Comparison of insulin sensitivity and islet β-cell function

	Control	Dapagliflozin 10 mg		P-value	
		Before	After	Con vs Bef	Bef vs Aft
Stumvoll 1st phase index	1342.43 ± 585.89	− 1032.85 ± 1017.65	− 963.63 ± 928.78	< 0.001	0.775
Stumvoll 2nd phase index	367.02 ± 164.83	− 150.13 ± 223.52	− 144.11 ± 271.71	0.001	0.920
HOMA-β	115.06 ± 50.61	56.02 ± 26.08	59.85 ± 32.77	0.019	0.428
HOMA-IR	2.27 ± 1.24	4.52 ± 2.27	3.78 ± 1.59	0.044	0.624
QUICKI	0.35 ± 0.023	0.31 ± 0.02	0.32 ± 0.02	0.019	0.381
ISIstumvoll	0.078 ± 0.038	0.009 ± 0.017	0.027 ± 0.014	0.001	0.060
ISIMatsuda	119.35 ± 79.74	54.45 ± 25.11	76.95 ± 54.69	0.097	0.093
AUC_Glu/Ins_	0.20 ± 0.17	0.55 ± 0.49	1.00 ± 1.53	0.446	0.327
AUC_Ins_	212.26 ± 175.00	145.76 ± 94.78	107.83 ± 66.60	0.402	0.069

Con, control; Bef, before; Aft, after; AUC_Glu_, area under the curve of glucose; AUC_Ins_, area under the curve of insulin; HOMA-IR, homeostasis model assessment of insulin resistance; HOMA-β, homeostasis model assessment of β-cell function; QUICKI, quantitative insulin sensitivity check index; ISIstumvoll, insulin sensitivity index proposed by Stumvoll et al.; ISIMatsuda, insulin sensitivity index proposed by Matsuda et al.; 1st, first; 2nd, second

### Correlations between changes in glucose levels and islet β-cell function

**Fig. 5:**
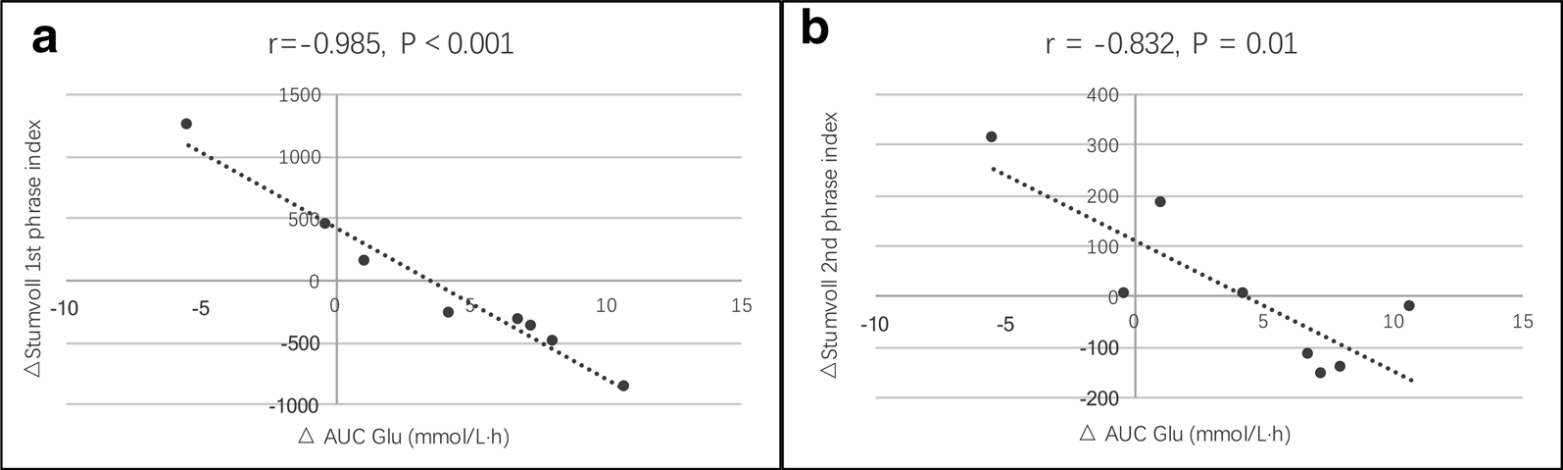
Correlation between changes in blood glucose and Stumvoll 1st (**a**) and 2nd (**b**) phrase indexes. The relationship between the changes was assessed using Pearson’s correlation coefficient. 1st, first; 2nd, second

The changes in glucose levels inversely correlated with changes in the Stumvoll 1st phase index (r = − 0.985, P < 0.01; Fig. 5a) and Stumvoll 2nd phase index (r = − 0.832, P = 0.01; Fig. 5b). However, there were no significant associations between the reduction of glucose levels and lipid parameters and insulin sensitivity parameters.

## Discussion

In this study, we explored the changes in SUA and 24-h UUA and FEUA levels before and after taking dapagliflozin 10 mg once daily for one week, as well as comparing the changes of parameters of insulin resistance and islet β-cell function before and after treatment. We found decreased SUA levels and increased FEUA levels after taking dapagliflozin, both with statistically significant differences. Additionally, there were improvements in glycemic control, insulin resistance and islet β-cell function but without statistically significant differences. We also found a positive correlation between the improvement of glycemic control and Stumvoll 1st phase index and Stumvoll 2nd phase index, which suggested the alleviation of glucotoxicity solely benefits islet β-cell function.

UA is the final metabolic product of purine compounds. Disorders of UA metabolism may cause hyperuricemia and gout. In recent years, a large body of studies have proved the clinical significance of UA in the development of various metabolic disorders, including T2DM [18–20]. On one hand, the prevalence of hyperuricemia in patients with T2DM is higher than that in subjects with NGT; on the other hand, hyperuricemia has been linked to both micro- and macrovascular complications in patients with DM [21]. Hyperuricemia can result from elevated UA production or reduced renal excretion. It was thought that hyperuricemia in patients with DM was dominated by reduced UA excretion because of decreased UA clearance and increased reabsorption caused by hyperinsulinemia [22, 23] and decreased GFR resulting from diabetic nephropathy.

Traditionally, parameters to evaluate the ability of the kidneys to excrete UA include 24-h UUA, clearance rate of UA, FEUA, excretion of UA per volume of glomerular filtration and UUA to urinary creatinine ratio, among which FEUA and 24-h UUA are subject to less of an impact from the eGFR. However, the level of 24-h UUA is affected by many factors, including dietary purine intake, amount of drinking water, urine output, renal function, and SUA. Some scholars recommend a more accurate and reliable index, FEUA, instead of 24-h UUA to quantify the level of UA excretion [24]. In our study, we found levels of both 24-h UUA and FEUA increased in subjects with T2DM at baseline. While the SUA levels were still higher than that of subjects with NGT, we speculate that the increased renal excretion of UA in urine might reflect an already compensatory mechanism of high SUA levels, or increased UA production might be the major cause of hyperuricemia in those patients with T2DM because increased oxidative stress resulting from T2DM and lipid peroxidation could lead to increased SUA levels, which act as endogenous antioxidants to protect the body [25]. Similar to our results, it was also reported in previous research that the mechanism of hyperuricemia is most likely due to overproduction of UA in certain patients with DM [26].

A variety of studies have reported reduced SUA levels after taking SGLT2 inhibitors. A post hoc analysis of prospectively collected data within the CANVAS program reported canagliflozin reduced serum urate concentrations and reduced events related to gout among patients with T2DM [3]. A meta-analysis of 62 randomized controlled trials involving 34 941 patients quantified the effect of any of the SGLT2 inhibitors (empagliflozin, canagliflozin, dapagliflozin, tofogliflozin, licogliflozin, or ipragliflozin) on reducing SUA levels in patients with T2DM [27]. However, in most previous studies, the reduced SUA levels were observed 12–26 weeks after treatment [28], and a few studies reported the excretion of UA could result in reduction of SUA in the first couple weeks [29]. Our study found the SUA-lowering effect of dapagliflozin can act within one week, and the level of SUA decreased significantly to nearly the same level of healthy subjects accompanied by a significant increase in FEUA, and there was a linear correlation between changes in the SUA and FEUA levels. The exact mechanism of the SUA-lowering effect of SGLT2 inhibitors is still unclear; the most widely accepted hypothesis is the possible involvement of the renal GLUT9 (SLC2A9) transporter, which is likely to play a predominant role in exchange of extracellular glucose for intracellular urate and act to enhance urinary urate excretion [30]. There are two GLUT9 transporter isoforms, which only differ in their N-termini: isoform 1 (GLUT9a) localized at the basolateral membrane and isoform 2 (GLUT9b) localized at the apical membrane of tubular cells. GLUT9b could also be found in the proximal tubule and in the collecting duct. Glycosuria resulting from SGLT2 inhibition causes an increased concentration of glucose in the proximal tubule, which could eventually lead to increased exchange of UA for reabsorbing glucose via GLUT9, while in collecting ducts, lesser glycosuria inhibits UA reabsorption via GLUT9 [30–33].

Reductions in insulin sensitivity and insulin secretion are the hallmarks of T2DM, and a large number of studies have revealed that hyperglycemia and hyperlipidemia are critical risk factors for islet β-cell dysfunction, which are known as β-cell glucotoxicity and lipotoxicity [6]. Currently, the beneficial effects of SGLT2 inhibitors on islet β-cell function have been reported. For example, canagliflozin [34] and dapagliflozin [35] were proven to improve hepatic and muscle insulin resistance, respectively. Empagliflozin-induced glycosuria improved islet β-cell function and insulin sensitivity [36]. However, a recent study using [18F]-fluorodeoxyglucose and positron emission tomography (PET) to measure tissue insulin sensitivity during the process of the hyperinsulinemic euglycemic clamp technique found no changes in tissue-level insulin sensitivity after treatment with dapagliflozin [37]. However, considering most of those studies used a relatively long period of treatment, and the improvement of islet β-cell function accompanied by the improvement in lipotoxicity achieved by loss of weight and amelioration in lipid metabolism, it was difficult to evaluate the individual effect of the alleviation of glucotoxicity on islet β-cell function. Thus, Shimo et al. [38] analysed C57BL/KsJ db/db mice treated for one week with 10 mg/kg/day empagliflozin, and they found expression levels of β-cell-related factors improved, such as MafA, insulin 1 and PDX1, from gene levels to protein levels, and the enhancement of β-cell proliferation was observed. However, the glucose-stimulated insulin secretion of isolated islets was not observed in the group treated with empagliflozin. They concluded that the alleviation of glucotoxicity for one week could alleviate expression levels of genes associated with islet β-cell function but was insufficient to achieve substantial improvement in islet β-cell function. In our study, a short period of treatment with dapagliflozin reduced glucose levels represented by the AUC_Glu_ according to 3-h OGTT, but without statistically significant difference, which indicated the emergence of obvious hypoglycemic effects requires more than one week of treatment. A certain degree of improvement in insulin sensitivity and β-cell function could be observed, but without statistically significant differences. On the one hand, we thought it was because the glucotoxicity had not been completely relieved, and there was no obvious difference in the improvement of islet β-cell function; on the other hand, it might be because the improvement in glucotoxicity in one week was not enough in humans to achieve obvious improvement in islet β-cell function, which was in accord with findings in animal experiments [38]. Furthermore, the linear correlation found between changes of AUC_Glu_ and parameters representing islet β-cell function, but not between glucose levels and insulin sensitivity parameters, showed improvement in islet β-cell function and was more related to improvement in glucose control after a short period of treatment. However, whether SGLT2 inhibitors can improve islet β-cell function by alleviation of glucotoxicity alone requires further research in larger sample populations and longer treatment periods.

In this prospective, pilot and exploratory study, in addition to hypoglycemic treatment, we found the effect of dapagliflozin on reducing SUA levels within one week. However, the sample size is small, and in-depth studies containing larger participants and different treatment period groups (including one-week, two-week, and four-week period groups) can be conducted to further explore the effects of treatment with SGLT2 inhibitors on the indicators of UA levels and islet β-cell function.

## Conclusions

In this study, after treatment with dapagliflozin 10 mg once daily for one week, we found dapagliflozin can reduce SUA levels by increasing UA excretion by the kidneys. Furthermore, a certain degree of improvement in islet β-cell function and the positive correlation between changes in glucose levels and islet β-cell function were observed without the interferences of changes in weight and lipid metabolism.

## Data Availability

The datasets generated during and/or analysed during the current study are available from the corresponding author upon reasonable request.

## Abbreviations

T2DM: Type 2 diabetes mellitus
SGLT2: Sodium-glucose cotransporter-2
SUA: Serum uric acid
PUMCH: Peking Union Medical College Hospital
HbA1c: Glycated hemoglobin
eGFR: Estimated glomerular filtration rate
MDRD: Modification of Diet in Renal Disease
DM: Diabetes mellitus
FBG: Fasting blood glucose
NGT: Normal glucose tolerance
ADA: American Diabetes Association
BMI: Body mass index
TC: Total cholesterol
HDL-c: High-density lipoprotein cholesterol
LDL-c: Low-density lipoprotein cholesterol
TG: Triglyceride
AUC_Glu_: Area under the curve of glucose
QUICKI: Quantitative Insulin Sensitivity Check Index
ISIMatsuda: Insulin sensitivity index proposed by Matsuda et al.
ISIstumvoll: Insulin sensitivity index proposed by Stumvoll et al.
AUC_Glu_/AUC_Ins_: The ratio of area under the curve of glucose and insulin
HOMA-IR: Homeostasis model assessment of insulin resistance
HOMA-β: Homeostasis model assessment of β-cell function
AUC_Ins_: Area under the curve of insulin
1st: First
2nd: Second
UUA: Urinary uric acid.
Una: Urinary sodium
UCl: Urinary chloride
FEUA: The fractional excretion of uric acid
FENa: The fractional excretion of sodium
FECl: The fractional excretion of chloride
OGTT: Oral glucose tolerance test
PET: Positron emission tomography
